# Brief results of heterotopic xenotransplantation of meningioma into the peritoneum of Wistar rats: an experimental study

**DOI:** 10.1590/1414-431X2025e15012

**Published:** 2026-03-02

**Authors:** L.F.M. da Silva, M.J.S. dos Santos, A.O. do Carmo, R.O.L. Silva, M.A.G. Campos, G.E.B. Silva, O.J. dos Santos, N. Salgado

**Affiliations:** 1 Hospital Universitário da Universidade Federal do Maranhão, São Luís, MA, Brasil; 2 Hospital da Universidade Estadual de Campinas, Faculdade de Ciências Médicas, Campinas, SP, Brasil; 3 Department of Emergency Medicine, School of Medicine, Duke University, Durham, NC, USA

**Keywords:** Meningothelial meningioma, Xenotransplantation, Peritoneal implant, Rats

## Abstract

Meningiomas are the most common primary intracranial tumors, and xenotransplantation models help to study their behavior and test therapies. This research developed a model implanting WHO grade I meningothelial meningioma into the peritoneum of immunosuppressed rats, comparing it to subcutaneous implantation. The primary objective was to analyze tumor growth and progression, focusing on the role of the microenvironment. Peritoneal implants (1.87±0.25 cm) grew significantly larger than subcutaneous implants (0.86±0.14 cm) (P<0.0001). An inverse correlation was found between weight variation and the difference in implant sizes, indicating that weight loss in animals was associated with larger implant growth. Animals that lost weight had significantly larger implants compared to those that gained weight. Implantation site and the animal's weight variation can significantly impact the growth of meningothelial meningioma fragments, with peritoneal implants showing greater growth and weight loss correlating with larger tumors.

## Introduction

Meningiomas are the most common primary intracranial tumors with an increasing incidence over time (1). Experimental models are critical for understanding the behavior of these tumors. Xenotransplantation models, known as “Avatar models”, provide opportunities for therapeutic testing, response evaluation, and guiding subsequent patient treatment (2). Implantation of tumor fragments reproduces the actual characteristics of a patient's tumor.

## Material and Methods

We developed a model using xenotransplantation of meningothelial meningioma (WHO grade I) in the peritoneum of rats immunosuppressed with mycophenolate mofetil (MMF) and compared with standard subcutaneous implantation. Our primary goal was to evaluate the growth and progression of meningiomas, examining the influence of the microenvironment on their development and tumor behavior.

Eight 60-day-old male rats underwent general anesthesia with ketamine and xylazine, followed by implantation of fresh meningothelial meningioma fragments measuring 1 cm in the major axis into the subcutaneous tissue of the right flank and the parietal peritoneum of the left abdominal wall. Subcutaneous fragments were implanted in a 3×2 cm pouch created after the dissection of the flank subcutaneous connective tissue; peritoneal fragments were implanted at 1.5 cm from the midline, and the tissue was sutured with 3 equidistant stitches of 5-0 mononylon suture.

**Figure 1:**
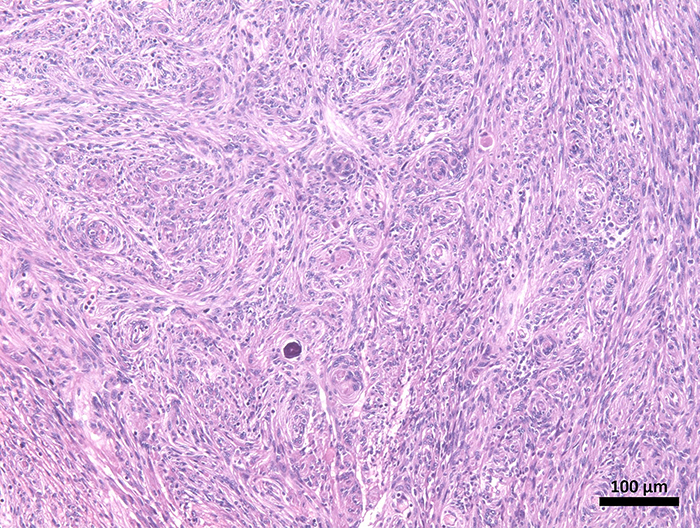
Meningioma fragment. Histopathological appearance of a meningioma tumor, showing an epithelioid spindle cell neoplasm with no marked atypia, mitotic activity, or necrosis, with foci of calcification and cells arranged in whorls (hematoxylin and eosin staining, scale bar 100 μm).

The xenografts were established using fresh human tumor fragments, not cell cultures. The tumor specimen was obtained from a patient undergoing surgical resection of a WHO grade I meningioma at the University Hospital of the Federal University of Maranhão (HUUFMA) (Figure 1).

Immediately after surgical excision, the tumor tissue was placed in sterile saline and transported to the experimental laboratory within the hospital complex. Fragments were prepared under sterile conditions without enzymatic digestion, dissociation, or *in vitro* culture to preserve the native microarchitecture and cellular integrity.

For the experimental duration of 28 days, MMF was administered orally by gavage as an immunosuppressant. The dosage was 40 mg/kg body weight, prepared as 40 mg/mL diluted in PBS. The gavage procedure utilized a curved 18GA, 39 mm stainless steel needle with a 1.2 mm diameter and a 2.25-mm distal protection sphere. Animals underwent two sequential surgical procedures: implantation of tumor fragments into the left parietal peritoneum and the right subcutaneous flank. Anesthesia was induced with ketamine (50 mg/kg) and xylazine (6.7 mg/kg) intramuscularly.

For the peritoneal cavity implantation, a fenestrated surgical drape technique was applied. A 3-cm midline skin incision was made in the caudal third of the abdomen using a #15 scalpel blade. The musculoaponeurotic plane was bluntly dissected to expose the linea alba. After grasping and elevating the linea alba, the peritoneum was incised and transected with scissors, allowing identification of intracavitary organs. A meningioma fragment was then transplanted onto the left ventral peritoneal wall, at the middle third of the incision, 1.5 cm lateral to the midline, and secured with three equidistant simple interrupted 5-0 mononylon sutures. The abdominal wall was closed in two layers using a simple continuous 5-0 mononylon suture, first in the musculoaponeurotic plane, then the skin (Supplementary Figure S1).

For subcutaneous implantation, the animal was repositioned in ventral recumbency. The epilated flank region was antiseptically prepared, and a fenestrated drape was placed. A 3-cm skin incision was made in the lower third of the right flank with a #15 scalpel blade. A 2-cm anterior blunt dissection was performed between the skin and aponeurosis, followed by the placement of the meningioma fragment onto the subcutaneous tissue. The incision was closed with simple interrupted 5-0 mononylon sutures (Supplementary Figure S2).

**Figure 2:**
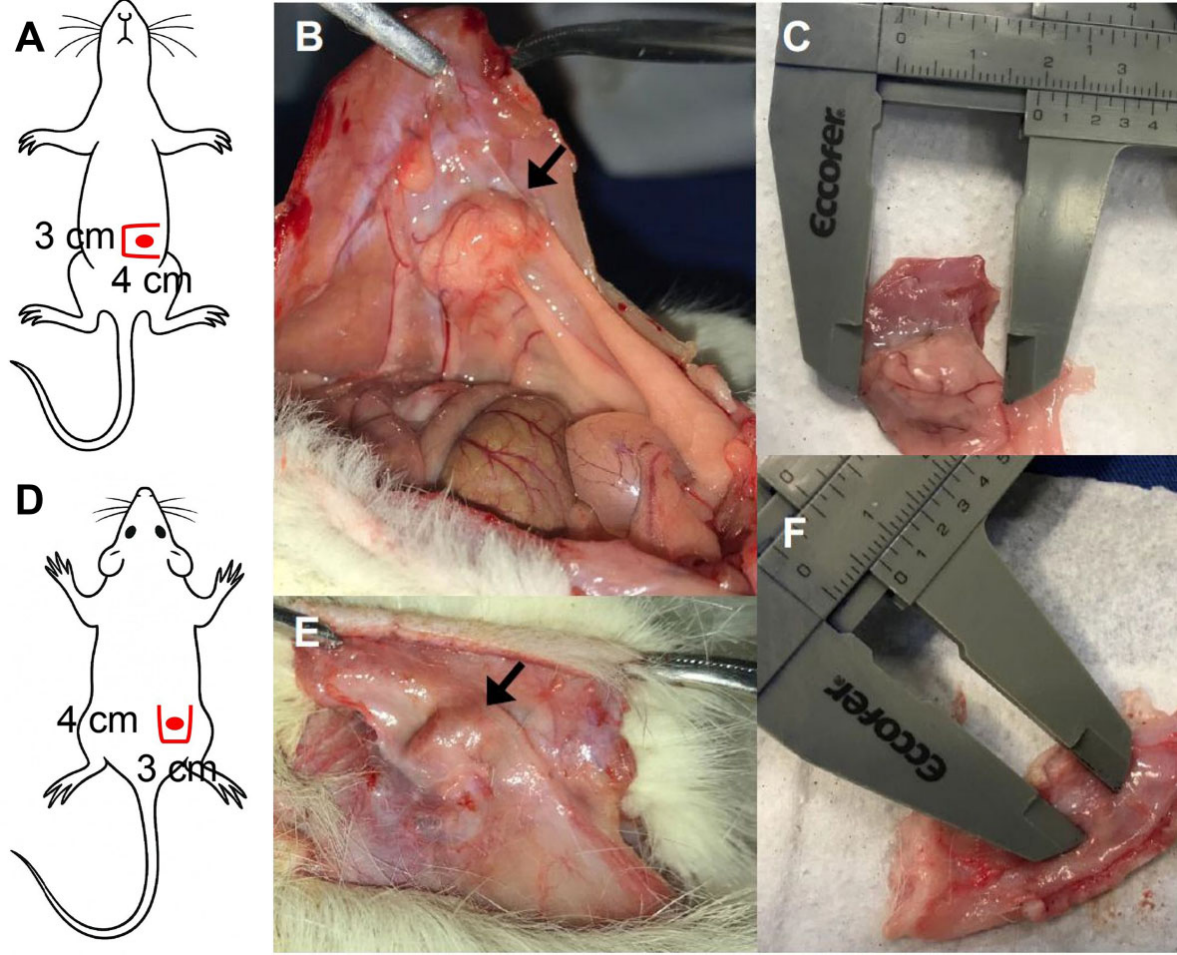
Explantation of the fragments. **A**, Diagram showing the incision for the peritoneal implant: a 3-cm book-style abdominal incision parallel to the midline and located 1.5 cm to the right of it, with two 4-cm perpendicular extensions at both ends (solid red lines), and the estimated location of the implant (red circle). **B**, Photograph of the peritoneal implant (arrow) showing evident neovascularization. **C**, Measurement of the longest axis of the implant using a caliper. **D**, Diagram showing the incision for the flank implant: a 3-cm book-style flank incision parallel to the previous incision and 1.5 cm posterior to it, with two 4-cm perpendicular extensions at both ends (solid red lines), and the estimated location of the implant (red circle). **E**, Photograph of the subcutaneous implant (arrow) with no macroscopic evidence of neovascularization. **F**, Measurement of the longest axis of the implant using a caliper.

After four weeks, animals were euthanized by an overdose of the same anesthetic agents: 2% xylazine (30 mg/kg) and 10% ketamine (180 mg/kg), administered to achieve respiratory arrest and complete absence of reflexes. Following death confirmation, surgical explantation was performed. Incisions were made 1 cm from the original surgical sites, opposite to the implant (right abdomen for the peritoneal implant, caudal flank for the subcutaneous implant). A 4-cm incision was created parallel to the initial surgical access, extended perpendicularly in a “book-leaf” fashion, enabling resection of the implant along with a 1.5-cm margin of the adjacent implantation site (skin or rectus abdominis muscle/peritoneum) (Figure 2).

The final length of the major axis (LMA) of both implants were compared using a paired sample *t*-test. Ethical approval was obtained from both the Institutional Animal Ethics Committee of UFMA (CEUA, Protocol #23115.009088/2018-71) and the Institutional Review Board for Human Research of Hospital São Domingos (CEP, Approval #3.493.153).

## Results

**Figure 3:**
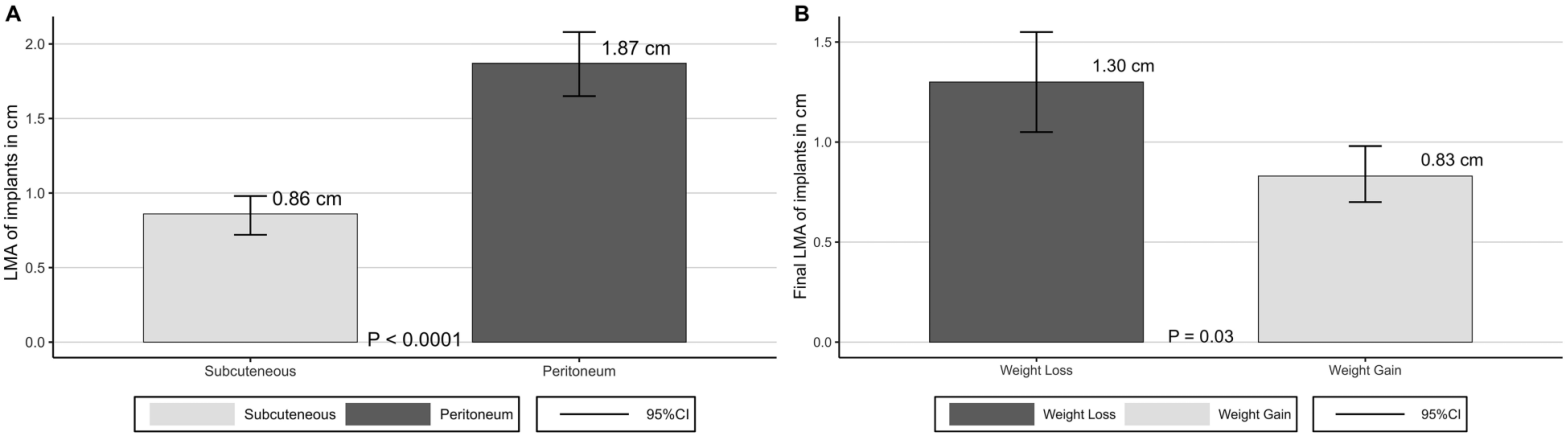
Final length of major axis (LMA) of implants by site of implantation (**A**) and by weight variation (**B**). Data are reported as means and SD; A, paired sample *t*-test; B, Student's *t*-test.

The LMA of peritoneal implants (1.87±0.25 cm, 95%CI: 1.66-2.08 cm) were greater than those of subcutaneous implants (0.86±0.14 cm, 95%CI: 0.74-0.98 cm). The difference between final LMA of the subcutaneous and peritoneal groups was inversely correlated with weight variation (final weight - initial weight) of animals (Pearson correlation coefficient r=-0.8 (P=0.0171)). Final LMA in animals with weight loss (1.30±0.24 cm, 95%CI: 1.05-1.55 cm) was higher than final LMA in animals with weight gain (0.83±0.18 cm, 95%CI: 0.70-0.96 cm), compared by Student's *t*-test (P=0.03) (Figure 3).

## Discussion

Although implants were 100% successful and viable in both sites, the mean LMA was 2 times higher in the peritoneal grafts. In addition, the difference in LMA at both locations was 1.5-times higher among rats that lost weight throughout the experiment. Both results attracted the attention of the authors.

This difference in growth may result from the anatomical differences between the two sites. Among its many specific functions in mammals, subcutaneous connective tissue allows the skin to slide over most parts of the body. It consists of layers of collagen fibers between the epimysium of the thin muscle adherent to the skin and the epimysium of the animal’s dorsal muscles. Transverse sections occasionally reveals tortuous vessels and nerves adapted for tissue mobilization (3).

The peritoneum, on the other hand, is the largest and most complex serous membrane of the body. It consists of a thin connective tissue layer, rich in nerves, vessels, and lymphatics. This greater vascular supply and larger surface area for tumor implantation may facilitate nutrient and oxygen diffusion to the tumor, promoting more robust growth. Also, the peritoneal environment may be more conducive for tumor growth due to the presence of ascitic fluid, which can contain growth factors and reduce mechanical constraints on expanding tumors (4). In addition, the peritoneum is believed to lack thrombogenicity (5). These functional and anatomical differences, including a richer vascular supply for graft nutrition, may provide a more favorable microenvironment in the peritoneum to allow implant growth.

Some possibilities should be considered regarding the inverse correlation with weight change. This may be due to the metabolic demands of the growing tumor mass in the peritoneum, which may lead to cachexia, a common paraneoplastic syndrome. Cachexia is characterized by weight loss, muscle atrophy, fatigue, weakness, and significant loss of appetite, which could explain the inverse relationship between tumor growth and weight variation (4). Obesity is increasingly associated with the incidence of tumors. A change in adipocytokine physiology is one pathway that may be responsible for this association (6). The expression of the leptin receptor (LEPR) correlates with the presence of leptin and has already been demonstrated in meningiomas. LEPR expression may play a role as a risk factor for meningioma growth (7). Higher leptin levels in some animals, and consequently reduced appetite, could lead to weight loss and higher growth of meningioma fragments due to LEPR-related pathway stimulation.

Another explanation for the association between weight loss and tumor growth is the Warburg effect, which suggests that even under aerobic conditions, glucose consumption for tumor replication occurs preferentially via glycolytic metabolism. The inverse association between hyperglycemia and meningioma occurrence has been demonstrated and attributed to this phenomenon (8). The predominance of energy metabolism by the glycolytic pathway has also been demonstrated in meningiomas (9). The Warburg effect is a conceptual framework for tumor metabolic demand, but not a mechanistic explanation for systemic glucose levels. Glycemia was not monitored, which constitutes a limitation of the present study. Another limitation is the experimental follow-up period. A minimum of 90 days of follow-up is recommended for animal models of meningioma (10).

The choice of the peritoneal cavity as the implantation site is based on clear biological and technical advantages. Specifically, the peritoneum provides a highly vascularized microenvironment, favoring enhanced neovascularization, superior oxygen and nutrient delivery, and improved graft viability. The peritoneal cavity induces a milder inflammatory response, reducing lymphocytic infiltration and lowering the risk of rejection compared to subcutaneous tissue (11,12).

This model is intended not to replace orthotopic intracranial models, but to serve as a complementary experimental platform that offers a practical approach for studying tumor growth dynamics, fibrosis, and neovascularization, and as a basis for preclinical therapeutic testing in settings where intracranial implantation is not feasible due to technical, ethical, or logistical constraints.

## Conclusions

These findings may help clarify the important metabolic pathways related to the growth and progression of meningiomas. The differences in growth patterns between peritoneal and subcutaneous xenotransplants highlight the importance of the tumor microenvironment in influencing tumor growth and behavior, which is a critical consideration in the development of preclinical models for cancer research.

We propose that future studies should incorporate glycemic, insulin, and lactate measurements to further investigate the metabolic impact of tumor growth in this model.

## Data Availability

All data generated or analyzed during this study are included in this published article.

## Supplementary Materials

Supplementary Material Click here to view [pdf].

## References

[1] Ogasawara C, Philbrick BD, Adamson DC (2021). Meningioma: a review of epidemiology, pathology, diagnosis, treatment, and future directions. Biomedicines.

[2] Onaciu A, Munteanu R, Munteanu VC, Gulei D, Raduly L, Feder RI (2020). Spontaneous and induced animal models for cancer research. Diagnostics (Basel).

[3] Kawamata S, Ozawa J, Hashimoto M, Kurose T, Shinohara H (2003). Structure of the rat subcutaneous connective tissue in relation to its sliding mechanism. Arch Histol Cytol.

[4] Andersen MS, Kofoed MS, Paludan-Müller AS, Pedersen CB, Mathiesen T, Mawrin C (2023). Meningioma animal models: a systematic review and meta-analysis. J Transl Med.

[5] Bonvini S, Albiero M, Ferretto L, Angelini A, Battocchio P, Fedrigo M (2012). The peritoneum as a natural scaffold for vascular regeneration. PLoS One.

[6] Avgerinos KI, Spyrou N, Mantzoros CS, Dalamaga M (2019). Obesity and cancer risk: emerging biological mechanisms and perspectives. Metabolism.

[7] Rutkowski R, Reszec J, Hermanowicz A, Chrzanowski R, Lyson T, Mariak Z (2016). Correlation of leptin receptor expression with BMI in differential grades of human meningiomas. Oncol Lett.

[8] Bernardo BM, Orellana RC, Weisband YL, Hammar N, Walldius G, Malmstrom H (2016). Association between prediagnostic glucose, triglycerides, cholesterol and meningioma, and reverse causality. Br J Cancer.

[9] Moreno-Sánchez R, Rodríguez-Enríquez S, Marín-Hernández A, Saavedra E (2007). Energy metabolism in tumor cells. FEBS J.

[10] Linsler S, Müller SJ, Müller A, Senger S, Oertel JM (2021). Fluorescence image-guided resection of intracranial meningioma: an experimental *in vivo* study on nude mice. Ann Anat.

[11] de Sousa WB, Garcia JB, Nogueira J, Furtado PG, dos Anjos JA (2015). Xenotransplantation of uterine leiomyoma in Wistar rats: a pilot study. Eur J Obstet Gynecol Reprod Biol.

[12] Wainwright DA, Horbinski CM, Hashizume R, James CD (2017). Therapeutic hypothesis testing with rodent brain tumor models. Neurotherapeutics.

