# Supplementary material for: Brief results of heterotopic xenotransplantation of meningioma into the peritoneum of Wistar rats: an experimental study
Source: Braz J Med Biol Res. 2026 Mar 2;59:e15012. doi: 10.1590/1414-431X2025e15012 (PMC12990303; doi:10.1590/1414-431X2025e15012)

Figure S1. Implantation of the fragment in the peritoneum. **A**, Diagram showing the shaved area (gray dashed lines), the 3-cm abdominal incision (solid red line), and the implantation site (red circle), located 1.5 cm from the midline. **B**, Incision of the skin and muscle layer along the linea alba with exposure of the peritoneal surface of the left abdominal wall. **C**, First fixation point of the peritoneal implant. **D**, Construction of a containment suture loop. **E**, Implant after placement of the three fixation knots. **F**, Closure of the muscle layer. **G**, Closure of the skin layer.

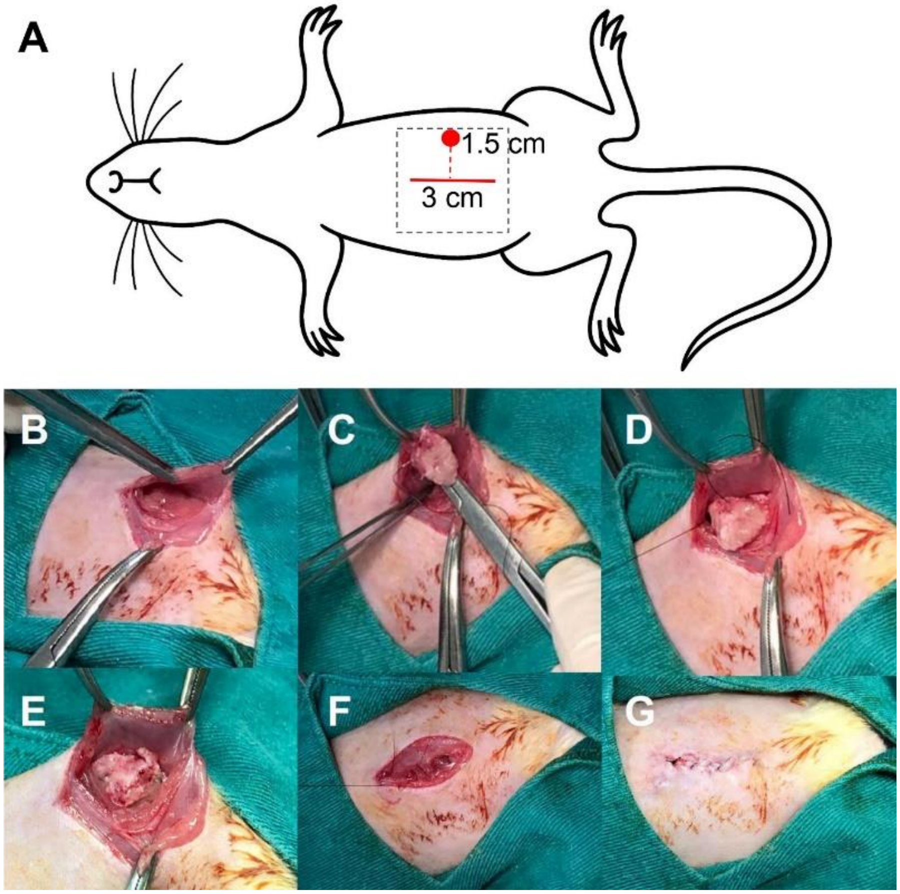

Figure S2. Implantation of the fragment in the subcutaneous tissue. **A**, Diagram showing the shaved area (gray dashed lines), the 3-cm flank incision (solid red line), the dissected area in the subcutaneous tissue (red hatched area), and the implantation site (red circle), located 1.5 cm from the midline. **B**, Skin incision on the right flank. **C**, Blunt dissection of the subcutaneous space. **D**, Exposure of the dissected space where the implant was placed, after which the skin was sutured.

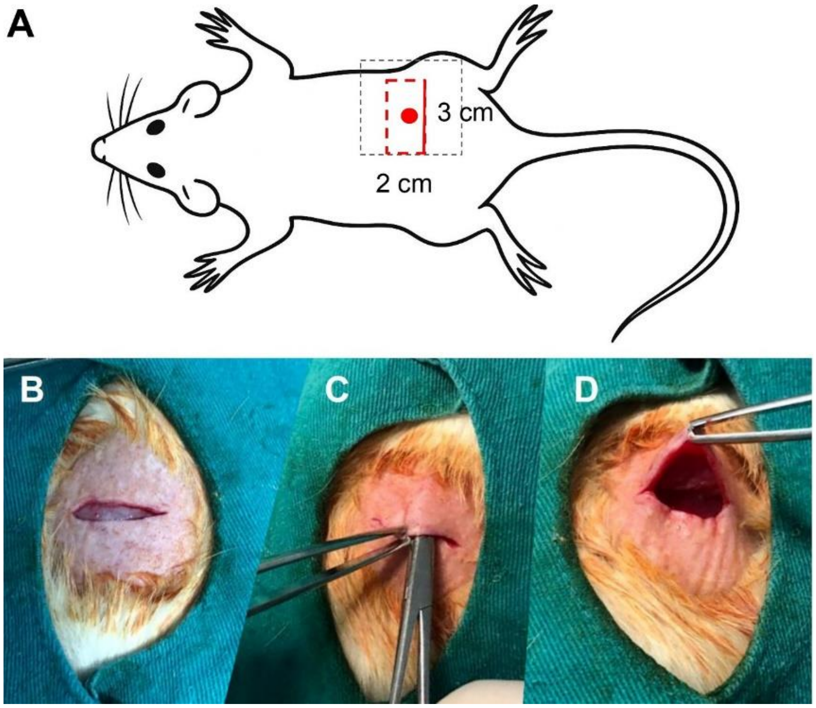

Supplement: Supplementary Material [file 1414-431X-bjmbr-59-e15012-suppl.pdf]
